# The Reddit cannabis subjective highness rating scale: Applying computational social science to explore psychological and environmental correlates of naturalistic cannabis use

**DOI:** 10.1371/journal.pone.0300290

**Published:** 2024-06-25

**Authors:** Meredith C. Meacham, Alicia L. Nobles, Carlton ‘CB’ Bone, Michael Gilbert, Johannes Thrul

**Affiliations:** 1 Department of Psychiatry and Behavioral Sciences, University of California San Francisco, San Francisco, California, United States of America; 2 Department of Medicine, University of California San Diego, San Diego, California, United States of America; 3 Department of Anthropology, Portland State University, Portland, Oregon, United States of America; 4 Independent, Portland, Oregon, United States of America; 5 Department of Mental Health, Johns Hopkins University Bloomberg School of Public Health, Baltimore, Maryland, United States of America; 6 Sidney Kimmel Comprehensive Cancer Center at Johns Hopkins University, Baltimore, Maryland, United States of America; 7 Centre for Alcohol Policy Research, La Trobe University, Melbourne, Australia; NYU Grossman School of Medicine: New York University School of Medicine, UNITED STATES

## Abstract

Social media data provide unprecedented access to discussions of active, naturalistic, and often real-time cannabis use in an era of cannabis policy liberalization. The aim of this study was to explore psychological and environmental correlates of cannabis effects by applying computational social science approaches to a large dataset of unprompted reports of naturalistic cannabis use with corresponding self-reported numerical ratings of subjective highness. Post title text was extracted via the Pushshift dataset from N = 328,865 posts to the r/trees Reddit community, where posters self-assess and disclose how high they feel on a scale from 1 to 10 (*M* = 6.9, *SD* = 1.8). Structural topic modelling and Linguistic Inquiry and Word Count (LIWC) dictionary-based approaches were applied to identify (1) frequently discussed topics and (2) text indicative of 5 psychological processes (affective, social, cognitive, perceptual, biological), respectively, as well as to examine relationships between subjective highness and (1) topic prevalence and (2) psychological process word counts. A 40-topic model was selected for interpretation based on semantic coherence and exclusivity. The most discussed topics in a 40-topic model were characterized by references to smoking places, social contexts, positive affect, cognitive states, as well as food and media consumed. In LIWC dictionary analyses, words mentioning affective, social, and cognitive processes were referenced more often than perceptual or body processes. Posters reported greater subjective highness when using language that referred to in-person social environments and lower subjective highness when using language that referred to online social environments and positive affect psychological states. This examination of unprompted online reports of naturalistic cannabis use identified textual content referring to affect and to other people as being associated with perceived effects of cannabis. These affective and social aspects of the cannabis use experience were salient to active posters in this online community and should be integrated into experience sampling methods and behavioral pharmacology research, as well as public health messaging.

## Introduction

The liberalization of cannabis policies worldwide since the early 2010s and related market expansion have catalyzed the rapid growth and diversification of cannabis and cannabinoid products [1], driving the need for information on risks and benefits of these products [2]. The U.S. Food and Drug Administration (FDA) has highlighted the importance of “real world data” to better understand the use and safety profiles of cannabis and cannabinoid products [3, 4]. Social media data provide unprecedented access to discussions of active, naturalistic, and often real-time cannabis use [5, 6]. Analyzing online discourse and communities is an avenue in which qualitative and computational methods have previously been applied to glean insights into cannabis and other substance-specific communities [7–13]. Multidisciplinary approaches in computational social science are needed to investigate and contextualize these large amounts of user-generated social media data [14, 15].

A construct of interest in cannabis science and regulation is acute subjective effect, “highness”, or “intoxication” level as reported by the person using cannabis [16–19]. This subjectivity contrasts with objective measurements of substance effects such as metabolite blood concentrations and heart rate. This subjective highness effect is pertinent to determining appropriate timing and dosages, as well as intoxicating and therapeutic effects, and the role of expectancies like placebo and nocebo effects [20–22]. Prior examinations of subjective effects mainly derive from behavioral pharmacological research, where participants in a controlled environment are administered a pre-determined dose and assessed for subjective and objective effects at regular intervals [17, 23, 24]. Importantly, a 2021 study demonstrated that assessment terminology for cannabis products or effects that are of limited face validity to participants can lead to specification error [25]. As measurements of subjective effects of cannabis are often adapted from measurements of effects of tobacco, alcohol, and other substances, there is a need for understanding the subjective effects of cannabis as reported by individuals in their everyday life to inform cannabis use assessment instruments as well as health and safety guidelines [26].

Reddit is a popular social media platform where user-generated text, link, and image content is submitted pseudonymously to topic-specific and volunteer-moderated discussion forums called subreddits. The subreddit r/trees is the largest cannabis community on the Reddit platform and the self-described, “go-to subreddit for anything and everything cannabis.” A systematic review of online “consumer-generated discourse” of cannabis identified extant research as primarily examining online data from Twitter, with less research from Reddit and other web-based forums [6]. The review notes that a limitation of much of this broad keyword-based social media and search-activity research is that mentions of cannabis may not represent actual use of cannabis.

We previously reported on an online community-created scale in r/trees where people include in their post how “high” they are on a scale of 0–10, indicated in brackets, and where 0 is “sober,” 1–2 is “buzzed,” and 10 is “in space” [S1 Fig]. In contrast to keyword-based social media data, people in the r/trees online setting who use this scale are inherently signaling recent or current use of cannabis. We found that mean subjective highness was significantly greater in posts mentioning high-THC dabbing, edible, and concentrate terms when compared to posts mentioning smoking terms [8]. However, posts that mentioned mode of use terms were relatively infrequent (17.7%); further exploration of the broader dataset may yield additional insights into naturalistic cannabis use experiences.

A key contribution to the understanding of psychoactive substances, both in academic literature and among people who use drugs, is the framework of set and setting. In this framework, the effects of psychoactive substances are dependent on the “set” of the person using the substance (e.g., internal beliefs, expectations, intentions) as well as their “setting” (e.g., environments that may be social, material, cultural). Although originally popularized from research on psychedelic substances, this framework has been applied to explain human responses to a range of psychoactive substances [27, 28]. For example, in 19^th^-century observations of hashish use by the members of the “Parisian Club of Hashischins” and 20^th^-century sociological essays like “Becoming a Marihuana User” the effects of cannabis are noted to be influenced by the physical environment and by sentiments of peer groups [29]. While most prior research with set and setting has focused on in-person settings, the application of this framework to online communities presents unique opportunities to understand cannabis experiences, especially given the role these online spaces and networks may play in shaping cannabis use experiences, expectations, and social norms [30]. In turn, this enhanced understanding may be used to connect research findings from controlled settings with experiences in “real-world” settings and improve the credibility and receptivity of cannabis risk messaging.

The aim of this study is to conduct an exploratory descriptive analysis of subjective highness ratings and corresponding text as reported in the r/trees community subreddit. Given the large volume of data available, we take a concurrent triangulation approach that combines two computational social science methods: structural topic modeling, a type of unsupervised machine learning that identifies themes in a set of documents [31], and Linguistic Inquiry and Word Count (LIWC), a text analysis tool that applies predefined dictionaries to capture expression of social and psychological states [32]. Overall, we seek to examine: What is the general discourse in the r/trees community when contributors post how “high” they are? How is this numerical “highness” rating related to the textual expressions of Reddit posters in r/trees?

## Materials and methods

### Data collection

Post titles to r/trees from January 2010 to October 2018, were downloaded in March 2019 from the Pushshift Reddit Dataset [33], resulting in approximately 2.5 million post titles. We extracted the numerical value from 1 to 10 for the subjective highness rating contained in brackets, parentheses, or braces using regular expressions in Python. Posts that did not have titles were therefore not included. Posts with subjective highness in the post body text but not the title were also excluded as the subreddit convention is to include the rating in the title and because the post body text could be long stories with many phrases unrelated to cannabis use. Post titles in posts with an image, link, or gif were included. This process yielded 336,541 unique post titles with subjective highness ratings of 1 to 10.

### Ethical considerations

This research was categorized as human subject exempt category 4 by the University of California San Francisco Institutional Review Board and consent was not required. r/trees is a public subreddit and findings are presented in aggregate. Unique usernames were tabulated using hashed anonymized strings. The authors did not have access to any other information that could identify individual participants. Sample quotations presented here are composites or are lightly reworded, and run through a search engine, to reduce the risk of re-identification. Although some social media research presents direct quotes to directly represent themes in the data, given the sensitive nature of this subreddit content and our naming of the subreddit, we elected to present paraphrased examples of themes [34].

### Approach

The computational approach for this textual analysis is inspired by the iterative, “computational grounded theory” approach developed by Nelson [35] that combines pattern detection using unsupervised computational analysis, pattern refinement with guided reading by human analysts, and pattern confirmation using supervised natural language processing methods. As we are not conducting grounded theory, we have adapted this approach to compare unsupervised machine learning (i.e., topic modeling) plus human annotation with a pre-defined dictionary method (i.e., LIWC). An overview of approach steps is presented in Table 1.

**Table 1:** Approaches to exploring relationships between textual content and subjective highness ratings.

Steps (software)	What/How	Statistical Inference
1. Data Extraction (Python)	336,541 post titles to r/trees with 1 to 10 in brackets from 2010 to 2018^1^	Numbers representing subjective highness are normally distributed (*M* = 6.9, *SD* = 1.8)
2.1 Structural topic modeling (R)	k = 40 topics,^2^ subjective highness as prevalence covariate	Association of topic prevalence with subjective highness
2.2 Human labeling of topics (Excel)	Representative 10 quotes/topic and sets of top 10 words/topic^3^ used to label topics	NA
3. Dictionary (LIWC software and R)	Linguistic Inquiry and Word Count (LIWC) of 5 psychological processes	Associations of psychological process word % with subjective highness (regression)
4. Comparison	Set and Setting Framework	NA

^1^. Preprocessing to 328,865 post titles (7,132 words)

^2^. Structural topic models were also run with k = 20 and k = 60 topics

^3^. Top 10 words by highest probability, FREX, Lift, Score

#### Structural topic modelling

Topic modelling is an automated computational method of analyzing large amounts of textual data and is a type of unsupervised machine learning [14, 36, 37]. By examining patterns of co-occurring terms within and across documents (e.g., social media posts), topic models computationally detect latent topics, and associate a probability of the topic being present in each document. Topics are defined as a set of words strongly associated with the probability of the word being present in the topic. Structural topic modeling extends this approach by allowing for the incorporation of an external covariate derived from document metadata such as timestamp or data source [31, 37]. In structural topic modeling, a given social media post (i.e., document) may be described by multiple topics.

Structural topic modeling for this analysis was conducted using the stm package v1.8.6 [31] in R Studio v1.2.5019. Pre-processing of the 336,541 extracted post titles included removal of words that appeared less than 15 times (the default), converting all letters to lower case, and removal of numbers, punctuation, and stopwords, yielding a corpus with 328,865 post titles and 7,132 unique words. Words were not lemmatized to increase later interpretability.

A series of three structural topic models were fit with k = 20, 40, and 60 topics. As there is no single “correct” number of topics and this number is user-specified, we selected these values of k based on a review of the literature employing topic modeling on short social media documents and the results of running the *searchK* function with 20 to 100 topics [S2 Fig]. These models were fit with the spectral initiation, given the large number of documents consisting of shorter lengths of text. The subjective highness rating was specified as a topical prevalence covariate with a continuous, normal distribution in each structural topic model. In the structural topic model with a topical prevalence covariate [38], the expected proportion of a document that belongs to a topic is a function of this prevalence covariate.

For each of these three models, the average exclusivity and average semantic coherence numerical scores were extracted and compared. [S3 Fig] For each topic, the first author (MM) also examined the top 10 words with highest probability and highest FREX score (which weights words by overall frequency and exclusivity to the topic) along with the top 10 post titles most representative of that topic to apply a first impression label to the topic. The 40-topic model was selected for further analysis given intermediate exclusivity and semantic coherence and the emergence of clear and unique topics distinct from the 20-topic model. While the 60-topic model included several new topics, many were variations on topics in the 20- and 40-topic models.

The expected topic proportions were output as the mean percentage that a given topic appears in the corpus of post titles, as each post title is represented by multiple topics. The relationship between expected topic proportion and subjective highness score as a linear regression coefficient with corresponding p-values were also output using the *effectEstimator* function. Topics where expected proportions were significantly associated with subjective highness score at *p* <. 05 were identified and plotted.

For each topic, the top 10 words according to four metrics (from the *labelThoughts* function) and top 10 post titles (from the *findThoughts* function) were exported into a separate file for human labeling, guided by deep reading of the post titles for each topic. The four metrics included highest probability words for a given topic, and three metrics that weight words by both overall and relative frequency in comparison to other topics (FREX, Lift, and Score). Based on these word lists and representative topics, the 40 topics were assigned topic labels independently by two authors familiar with the Reddit platform and substance use terminology (MM, MG). These two authors then compared, discussed, and converged on topic labels for 33/40 topics and grouped similar topics into categories. For the remaining 7 topics, we were not able to identify clear and consistent topic labels based on both top words and posts, and so these topics were not examined further [39]. The 33 labelled topics were then assigned into one of 11 more comprehensive categories or groups, as is common for models with many topics [40–42].

#### LIWC dictionary

We then applied a previously developed dictionary to determine the frequency of words with specific meanings and then compared these findings with those of structural topic modeling. Linguistic Inquiry and Word Count (LIWC) [32] is a rule-based dictionary and software developed by psychologists to generate normalized counts of words in textual data that fit into linguistic and psychological process domains [43]. The dictionary has been validated in hundreds of studies. LIWC can output over 100 text-based analysis variables organized into over-arching categories that include linguistic dimensions, grammar, and psychological processes. For more in-depth information and history, see https://www.liwc.app/.

We selected the five psychological process constructs captured by LIWC (affective, social, cognitive, perceptual, biological) to examine in relation to the subjective highness rating. Within affective processes, positive emotion and negative emotion sub-constructs were also selected, given their opposing valences. We note that while negation words such as “not” are available as a LIWC variable, as is a proprietary assessment of tone, some meanings of phrases like “not happy” may be missed in this word-by-word approach.

Word counts from LIWC software were imported into R software and mean percent of words indicating a given process was calculated across all subjective highness ratings. Bivariate relationships between the mean percent of words for a given process and subjective highness rating were estimated using linear regression analyses, with the percent of words referring to given psychological processes as a function of subjective highness rating. These dictionary-based patterns were then compared with patterns detected in the structural topic modeling of steps 1 and 2, and further interpreted through the set and setting framework.

## Results

From 2010 to 2018, there were 336,541 unique post titles with 1–10 in brackets indicating a self-reported subjective highness rating, representing approximately 13% of all posts during this period. These numbers had a normal distribution (mean = 6.9, SD = 1.8). There were 117,286 distinct usernames who contributed these posts, but ratings and text content were treated as unique for this analysis given differences in context for each reported rating instances.

### STM topic prevalence & associations with subjective highness rating

In the 40-topic model, the expected topic proportion, or prevalence, ranged from 0.8% to 8.6% across the corpus of post titles. The 20 most-discussed topics with labels, categories, and top words are presented in Table 2. (The remaining labelled topics are presented in S1 Table) The most discussed topic (“smoke sessions”) was characterized by references to places where the poster smokes, often on a regular basis. *“Whether you’re smoking on your front porch*, *living room*, *on a mountain*, *inside your room*, *or your buddy’s backyard on the other side of the world*, *I hope you have a great smoke*! [7].*”* The next most discussed topic (“eager sharing of stories”) was characterized by posters sharing an observation or story, with a sense of eagerness and excitement: “*guys guys guys*! *…*. *just had the best high shower thought while literally in the shower* [8].

**Table 2:** Topic prevalence, descriptions, and relationship to subjective highness rating.

#	Mean Topic Proportion / Prevalence	Topic Label	Category	Top Words	Topic Description	Relationship to Subjective Highness^1^
1	8.6%	smoke sessions	location	today, day, weed, smoke	Reference to place where poster smokes, often on a regular basis	-0.0016***
2	7.7%	eager sharing of stories	social	just, guys, thought, something	Sharing an observation or story, with a sense of eagerness	-0.0004*
3	7.1%	just / recently	time	made, smoked, got, realized	Characterized by recent event, use of "just"	0.0004*
4	4.7%	meta-cognition	cognition	high, get, youre, know	Introspective thoughts, indicating self-awareness and self-questioning	0.0002
5	4.1%	other people	social	friend, said, solid, buddy	Reference to friends, family, acquaintances, strangers	0.0010***
6	3.7%	last night	time	night, last, shit, got	Mentions of "last night"	0.0009***
7	3.5%	mellow positivity	affect	good, perfect, song, way	Positive satisfaction or satiation, low state of arousal	-0.0004*
8	3.4%	abstract observations	cognition	like, looks, blue, dream	General but abstract observations about life and cannabis	0.0003
9	3.2%	camaraderie	social	ents, fellow, right, now	Signaling or seeking community and solidarity with subreddit members	-0.0005**
10	3.1%	analytical thoughts	cognition	life, real, cant, talk	Philosophical, analytical, articulated observations	0.0000
11	3.1%	media 1	media	watching, watch, walking, show	TV shows, movies, videos	0.0001
12	3.0%	food 1	food	making, eating, cake, munchies	Foods prepared or ordered and eaten	-0.0001
13	2.7%	media 2	media	movie, time, watching, watched	TV shows, movies, videos	0.0002
14	2.4%	media 3	media	watching, looking, park, earth	TV shows, movies, videos	0.0001
15	2.3%	smoke spots	location	smoke, spot, todays, new	Current location where poster is smoking	-0.0004**
16	2.3%	product combos	preparation	bowl, kief, just, keif	Combinations of products, often with kief	0.0001
17	2.2%	playing games	games	playing, suddenly, like, pokemon	Video and board games	0.0000
18	2.1%	chill vibes	affect	morning, good, happy, self	Calm demeanor, sometimes referencing time of day or day of week	-0.0002
19	2.1%	first time posting	social	first, time, post, long	Signaling first post, often in self-deprecating or hedging manner	0.0003*
20	2.1%	laughter	affect	laughing, almost, stop, getting	Uncontrollable laughter in reaction to something; high state of arousal	0.0000

^1^.

* p < .05,

**p < .01,

*** p < .001

Overall, the most discussed topics were characterized by references to smoking locations, social contexts, time, affect, and cognition. Food and media consumed (e.g., movies, video games, tv shows) were other common topic categories. There were few topics characterized by specific product type or route of administration. We did not observe any topics characterized by motivations for recreational or medicinal use or referring to experiences of adverse effects.

**Fig 1:**
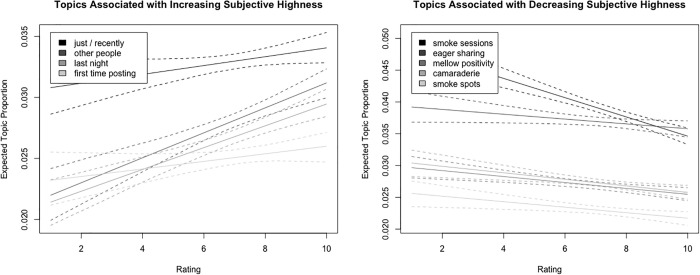
Relationships between subjective highness rating and topic prevalence.

Expected topic proportion was significantly associated with subjective highness rating for 9/40 topics (*p* < .05) [Fig 1]. Four topics were discussed *more* often as subjective highness increased. These included two topics alluding to social environments. The topic with the strongest positive association with subjective highness was characterized by in-person social contexts (“other people”), often relating humorous stories: “*I was in my friend’s car as a passenger in the Dunkin Donuts Drive Thru and asked*, *“do you sell donuts here*? [8]” Another topic (“first time posting”) signaled a poster’s entrance to creating content for this online community: “*Longtime lurker*, *first time poster*. *At a* [9] *and found this funny post*. *I added a drawing*.*”* Two other topics referred to time frame (“just/recently”, “last night”): *I ate a brownie an hour ago and just realized the word "landing" (as in landing a plane) literally means "landing" the plane* [5]. “Just” in these posts may not refer to cannabis use per se, but to some other occurrence after cannabis consumption. When people posted about “last night,” they sometimes indicated that they had been too high to post at the time: *“Got to a* [9] *last night and woke up to this on my phone*. *Too far gone to post but last night was a good night*.*”*

Five topics were discussed *less* often as subjective highness numbers increased. Again, two topics were characterized by social environments, but in online settings (“eager sharing of stories”, “camaraderie”): *“Fellow ents*! *I propose a musical experience—pack a bowl and click this link at a* [5] *and over*.” (“Ents” is how people in r/trees refer to each other and is a reference to the tree-like creatures in the Lord of the Rings.) Another topic in this group was characterized by positive affect (“mellow positivity”): “*Have a listen with me to this perfect Saturday morning song to set a good mood*, *at a* [6].*”* Two other topics were characterized by place or location (“smoke sessions” and “smoke spots”): “*Today’s backyard smoke spot by the river* [4]*”*. These posts often included photos of a joint or pipe and an outdoor setting when viewed in the full Reddit context via web browser or mobile app.

In sharing these sample posts, we also note that each post could contain several topics. For example, *“First time posting*, *long time lurker*, *my fellow ents*. *I got to a* [9] *last night with my roommate and drew this picture*. *I just realized how nice our smoke spot is”* is characterized by topics of “first time posting,” “camaraderie,” “last night,” “other people,” “just/recently,” and “smoke spot.”

### LIWC dictionary term prevalence & associations with subjective highness rating

**Fig 2:**
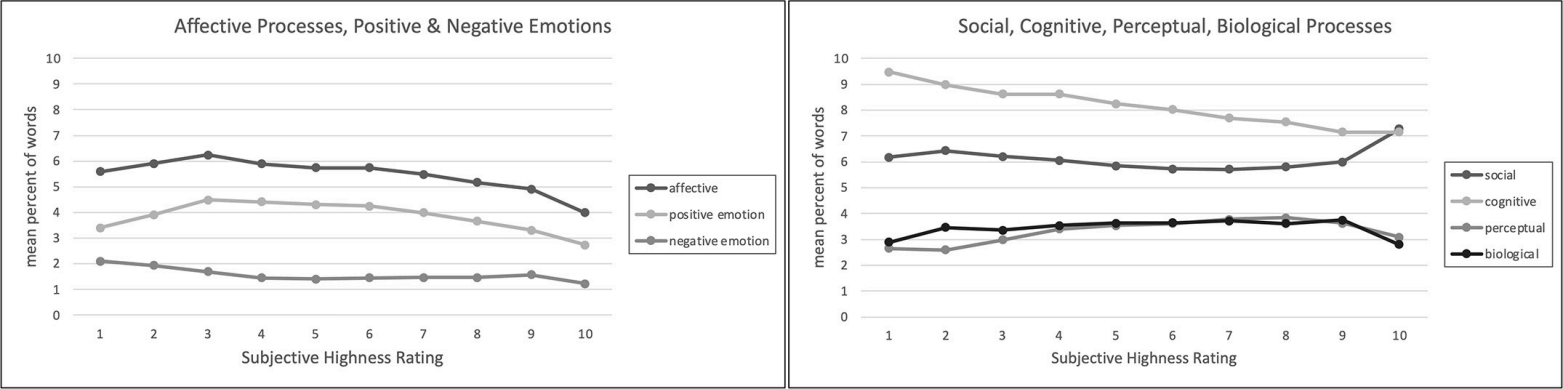
Plots of mean percent of words indicating LIWC psychological processes.

In LIWC dictionary analyses, the mean percent of words referring to psychological process categories was greatest for cognitive processes (7.8%), followed by social (5.9%) and affective (5.4%) processes, and then perceptual and biological processes (both 3.6%). Within affective processes, the occurrence of positive emotion words (3.9%) was greater than the occurrence of negative emotions words (1.5%) (Table 3). Mean percent of words referring to these processes is plotted along with subjective highness in Fig 2.

**Table 3:** LIWC dictionary psychological processes word occurrence and relationship to subjective highness rating.

Psychological Process	Mean % of words across all post titles	Relationship to Subjective Highness ^a^	Sample words from LIWC2015 Manual
affective	5.4%	-0.26***	happy, cried
positive emotions	3.9%	-0.24***	love, nice
negative emotions	1.5%	-0.02***	hurt, ugly
social	5.9%	0.10***	mate, talk
cognitive	7.8%	-0.25***	cause, know
perceptual	3.6%	0.03***	look, heard
biological	3.6%	-0.05***	eat, blood

^a^. Linear regression coefficient,

*** *p <* .*001* for all estimates

In linear regression analyses, social and perceptual processes words were associated with increasing subjective highness—as reported subjective highness increased, the percent of words referring to these processes also *increased*, with the strongest relationship for social processes (Β = .10). For each 1 unit increase in subjective highness rating, the percentage of social process words increased by.1%, 1.0% over the whole scale. Affective, cognitive, and biological processes were inversely associated with subjective highness—as reported subjective highness increased, the percent of words referring to these processes *decreased*. This relationship was strongest for affective (Β = -.26) and cognitive (Β = -.25) processes and, within affective processes, for positive emotions (Β = -.24). See Table 3.

We note some non-linear observations at the upper and lower ends of this rating scale. For example, the mean percent of affective words increases slightly from 1 to 3 and then decreases, a pattern also observed for positive emotions. For social process words, the decreasing relationship from 2 to 9 reverses direction for ratings of a 10. Similarly, while perceptual and biological process words slightly increase with subjective highness, they both decrease from 9 to 10. In quadratic regression models, there was a significant quadratic effect for all processes except for cognitive processes and negative affect, which corresponds to observed trends in Fig 2.

### Comparing topic modeling and dictionary findings

Comparative findings from structural topic modeling and the LIWC dictionary approaches are summarized in Table 4. As subjective highness rating increases, the “other people” topic and LIWC dictionary social processes were discussed more often, demonstrating concordance in the increasing association between subjective highness and references to in person social context settings. Additionally, as subjective highness rating increases, the “mellow positivity” topic and LIWC affective processes (mainly positive emotion) were discussed *less* often, demonstrating concordance in the decreasing association between subjective highness and references to affect. Although cognitive processes words in the LIWC approach were mentioned less often with increasing subjective highness, the prevalence of cognitive topics were not significantly associated with subjective highness rating in our structural topic modeling approach.

**Table 4:** Comparison of approaches demonstrating relationships between subjective highness rating and textual content.

	Approach: Structural Topic Modeling	Approach: LIWC Dictionary
***Linear Relationships***	***Textual Content of Post Titles***	
**Increasing/positive/proportional association**	Social—offline other people (topic #5)	Social—offline social process words
**No observed statistical association**	Cognition meta-cognition (#4), analytical thoughts (#10) Activities food (#12), games (#17), media (#11, 13, 14)	
**Decreasing/negative/inverse association**	Affect mellow positivity (#7) Social—online eager sharing of stories (#2), camaraderie (#3) Place smoke sessions (#1), smoke spots (#15)	Affect affective process words Cognition cognitive process words

## Discussion

In this study of over 300,000 self-reports of subjective highness ratings posted to the r/trees subreddit 2010–2018, we applied structural topic modeling and the LIWC dictionary to examine the overall discourse of these posts, as well as the relationship between these numerical values and corresponding textual content. During the examined period, over 1 in 10 posts to the subreddit r/trees contained a subjective highness rating, which reflects that this measurement convention was widely adopted. Cannabis effects reported in controlled laboratory conditions may differ from those experienced in naturalistic settings. For this “real world” dataset of experiences in naturalistic settings, we may not know the exact timing or dose of cannabis or THC [44], but we can infer that the posters had an experience that was remarkable or salient enough to share with this online community.

We found that the most prevalent topics referred to both psychological sets (cognition and affect) and environmental settings (smoking locations, social contexts). Within these posts to r/trees, there were few topics characterized by modes of use and no topics characterized by medical motivations or adverse effects from cannabis use. In addition to examining the overall discourse of these post titles, we also examined the relationship between subjective highness rating and corresponding text. In general, the higher the rating, the more likely posters referred to the recent past and to in-person social contexts. The lower the rating, the more likely posters referred to cognitive and affective processes, online social contexts, and smoking sessions or spots. In comparing the structural topic modeling and LIWC dictionary findings, we observed concordance of a positive relationship between subjective highness rating and references to social settings. This primarily refers to in-person social topics and social process words [45], whereas social context topics that referred to the online community of r/trees were discussed more often at lower levels of subjective highness.

This finding of greater subjective highness reported in in-person social settings has several potential interpretations. First, around others, people may use more in quantity than they otherwise would, which has also been reported with alcohol [46]. In social settings, people may be more likely to use cannabis with variable potency or other quality information [44, 47]. Additionally, as with other substances people may also become more aware of how high they are when interacting with other people. Implications for public health risk communication include messaging around situations where people may get “too high” unexpectedly [48]. A related implication is the role social environments have on self-titration to an optimal level of experience, while also minimizing harms to oneself or others (such as developing a cannabis use disorder or driving while intoxicated). Prior research has found that while use of higher potency cannabis is correlated with greater intoxication, individuals may intentionally consume less in certain settings [16]. An online survey study found that intoxication level perceived as safe for driving was associated with frequency of driving under the influence of cannabis, while typical level of intoxication was not [18].

We also observed concordance in structural topic modeling and LIWC findings in the inverse relationship between subjective highness rating and affect. Findings from the present analysis indicate that expression of affect or emotion was more likely to be positive than negative. Positive sentiment was similarly noted to be more common than negative sentiment in a systematic review that examined sentiment of consumer-generated cannabis content [6]. With respect to public health messaging, an implication of these findings of positive tone and no adverse effects topics is that social media perceptions of cannabis tend to be positive; overly negative or cautious messaging may not be seen as credible [48, 49].

While positive affect mentions seemed to decrease as subjective highness increased overall, we also note a non-linear trend in LIWC analyses. This has implications for potency and dosing information in that the effects of cannabinoid products are non-linear. For example, recent research has found that cannabis may provide anxiety reduction at lower doses of THC but increase anxiety at higher doses [50]. Existing research using experience sampling methods, which investigate the effects of cannabis use systematically in an individual’s real-world environment, has produced inconsistent findings regarding positive and negative affect. A review of the literature found no consistent associations between cannabis use and affect in community samples [51]. However, a more recent Ecological Momentary Assessment (EMA) study reported that amount of cannabis used was associated with higher positive affect [52]. Another EMA study found that cannabis use and negative affect were moderated by social context [53].

The decline in expression of affective topics and of affective and cognitive process words as subjective highness increases also coheres with the stated meaning of the highness scale as implying the person using cannabis is becoming less grounded to reality and “higher.” A research implication is to keep “highness” in Visual Analog Scales and other cannabis rating scales as a meaningful and brief measure that may reduce cognitive burden and be a familiar construct [54].

There are several potential areas of future research following this exploratory analysis. Further analyses could extract frequently occurring markers of time (e.g., “last night”) to filter the recency of these subjective highness reports. Other methods not applied here include modeling of n-grams, or sequences of words, and word embeddings. While the text data in the present analysis did not include sufficient mentions of potency or dosing, more recent consumer generated discourse could be examined. Expressions of subjective highness and effects of cannabis likely differ in other cannabis-related subreddits; for example, in subreddits where people share strategies and challenges in reducing or stopping use of cannabis [10], discuss other cannabinoids like delta-8 THC [55], or discussed access to and experiences with medically indicated or motivated cannabis use.

There are several limitations to this research. One limitation is that these data were only examined through 2018, prior to the legalization of cannabis in many U.S. states and rapid expansion of access to modes of use like concentrates and edibles and to novel cannabinoids. On the other hand, a strength of this approach is that during this timeframe we can infer that the mode of use was primarily smoking of delta-9 THC and that we captured the years when this subjective highness in brackets convention was used most often. Additionally, we do not know who the posters are demographically or where they are geographically, though Reddit traffic usage indicates they were likely in the United States [56]. Computationally, there are many other specifications of the structural topic model that are possible, including other numbers of topics in the model. For the LIWC dictionary, a 2022 update includes mappings of language commonly used on social media.

This subjective highness rating reflects online community-driven efforts to create a measurement convention for relating shared experiences of cannabis consumption. Our examination of these online reports of naturalistic cannabis use identified textual content referring to affect and to other people as being associated with perceived effects of cannabis. These affective and social aspects of the cannabis use experience were salient to active posters in this online community and should be integrated into EMA and behavioral pharmacology research as well as public health messaging.

## Supporting information

S1 Fig Highness chart from r/trees posted in 2012. (TIFF)

S2 Fig Diagnostic values by number of topics. (TIFF)

S3 Fig Semantic coherence vs. exclusivity for 20, 40, 60 topics. (TIFF)

S1 Table Continuation of table 2 for topics 21–40 with labels. (DOCX)
