# Supplementary figures and images for: The Reddit cannabis subjective highness rating scale: Applying computational social science to explore psychological and environmental correlates of naturalistic cannabis use
Source: PLoS One. 2024 Jun 25;19(6):e0300290. doi: 10.1371/journal.pone.0300290 (PMC11198820; doi:10.1371/journal.pone.0300290)

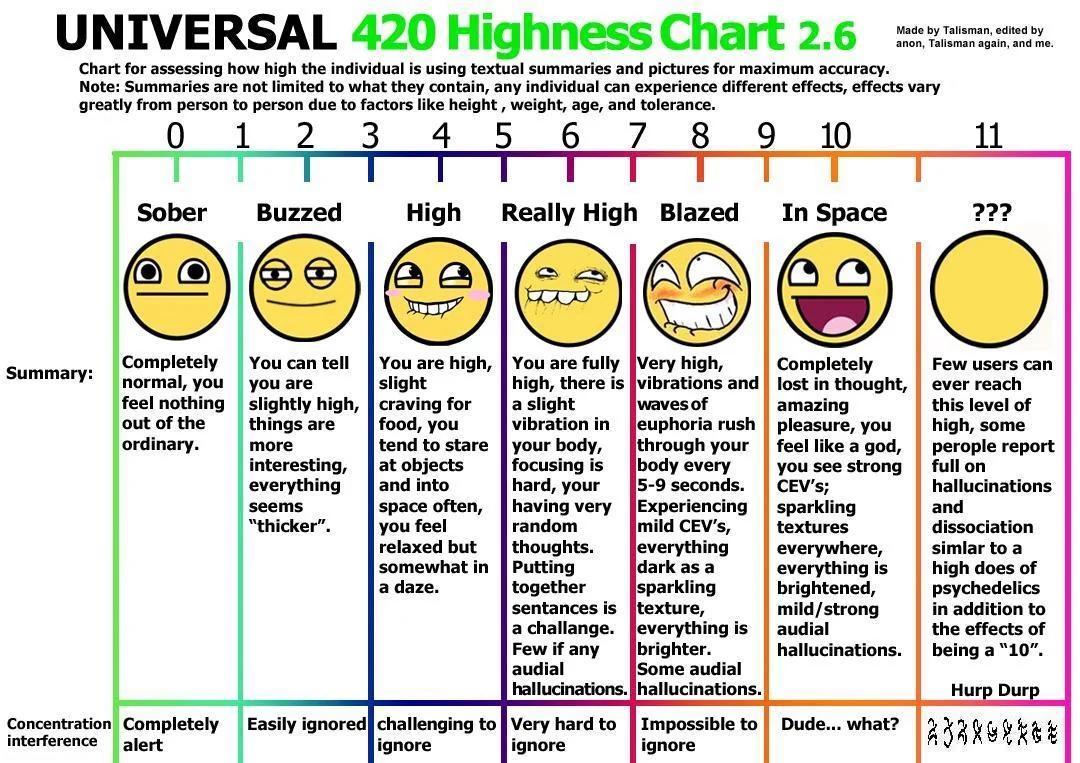

Supplement: S1 Fig — (TIFF) [file pone.0300290.s001.tiff]

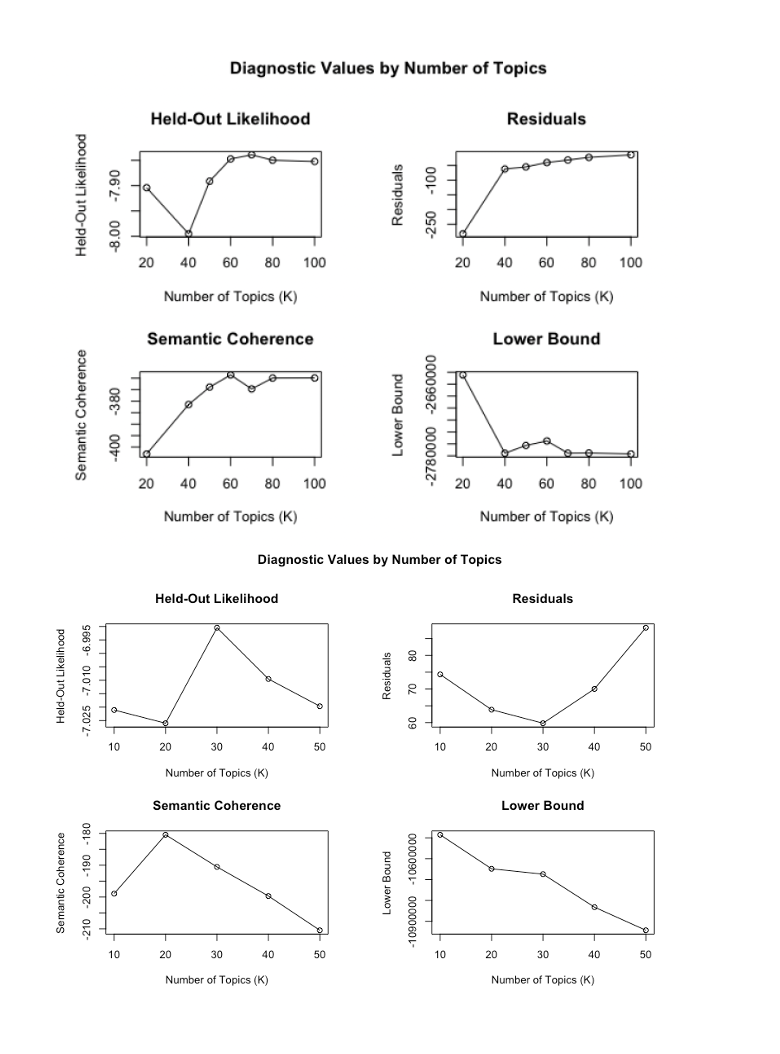

Supplement: S2 Fig — (TIFF) [file pone.0300290.s002.tiff]

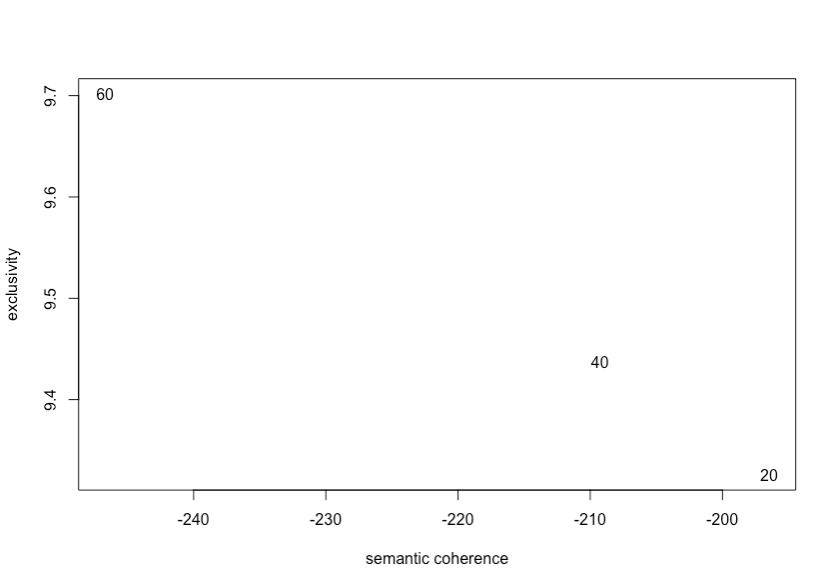

Supplement: S3 Fig — (TIFF) [file pone.0300290.s003.tiff]
