# Supplementary material for: The Reddit cannabis subjective highness rating scale: Applying computational social science to explore psychological and environmental correlates of naturalistic cannabis use
Source: PLoS One. 2024 Jun 25;19(6):e0300290. doi: 10.1371/journal.pone.0300290 (PMC11198820; doi:10.1371/journal.pone.0300290)
Supplement: S1 Table — (DOCX) [file pone.0300290.s004.docx]

**S1 Table 1. Continuation of Table 2 for topics 21-40 with labels.**

| **#** | **Mean Topic Proportion / Prevalence** | **Topic Label** | **Category** | **Topic Words** | **Topic Description** |
| --- | --- | --- | --- | --- | --- |
| 21 | 1.8% | food 2 | food | cheese, bacon, new, made | Various foods prepared or ordered and eaten |
| 22 | 1.7% | playing video games | games | play, game, playing, video | Names of video games |
| 23 | 1.7% | nature | activities | love, trees, gotta, like | Spending time in nature or mentioning plants and animals |
| 24 | 1.7% | food 3 | food | sweet, water, like, jesus | Various foods prepared or ordered and eaten |
| 26 | 1.6% | body awareness | cognition | feel, like, teeth, dancing | Mentions unusual awareness of body, like brushing one's teeth or feeling dry mouth |
| 31 | 1.2% | does anyone else | social | else, anyone, like, beer | Asking "does anyone else?"; Seeking confirmation of experience |
| 32 | 1.2% | wake and bake | events | wake, bake, first, morning | Reference to behavior of smoking in the morning, often before eating breakfast |
| 35 | 1.1% | food 5 | food | guy, good, style, baking | Various foods prepared or ordered and eaten |
| 36 | 1.0% | activities | activities | fixed, rain, chair, just | References various household chores and general activities |
| 37 | 1.0% | food 6 | food | pizza, ultimate, just, nom | Various foods prepared or ordered and eaten |
| 38 | 0.9% | media 4 | media | new, first, just, breaking | Names of TV shows, movies, online videos |
| 39 | 0.9% | holidays and celebrations | events | birthday, just, ents, like | References to using cannabis on birthdays, holidays, and other celebrations |
| 40 | 0.8% | timebomb | preparation | first, just, like, timebomb | Preparation of joint in a bowl |
